# Identifying rare variants from exome scans: the GAW17 experience

**DOI:** 10.1186/1753-6561-5-S9-S1

**Published:** 2011-11-29

**Authors:** Saurabh Ghosh, Heike Bickeböller, Julia Bailey, Joan E Bailey-Wilson, Rita Cantor, Robert Culverhouse, Warwick Daw, Anita L DeStefano, Corinne D Engelman, Anthony Hinrichs, Jeanine Houwing-Duistermaat, Inke R König, Jack Kent, Nan Laird, Nathan Pankratz, Andrew Paterson, Elizabeth Pugh, Brian Suarez, Yan Sun, Alun Thomas, Nathan Tintle, Xiaofeng Zhu, Andreas Ziegler, Jean W MacCluer, Laura Almasy

**Affiliations:** 1 Human Genetics Unit, Indian Statistical Institute, Kolkata 700018, India; 2 University of Göttingen Medical School, Göttingen, 37073, Germany; 3 Department of Epidemiology, David Geffen School of Medicine, University of California, Los Angeles, CA 90095 USA; and Research Service, VA GLAHS Epilepsy Center of Excellence, Epilepsy Genetics/Genomics Laboratories, Los Angeles, CA, USA; 4 National Human Genome Research Institute, National Institutes of Health, Baltimore, MD 21224, USA; 5 Department of Human Genetics, David Geffen School of Medicine, University of California, Los Angeles, CA 90095, USA; 6 Department of Internal Medicine, Washington University School of Medicine, St Louis, MO 63110, USA; 7 Division of Statistical Genomics, Washington University School of Medicine, St Louis, MO 63110, USA; 8 School of Public Health, Boston University, Boston, MA 02118, USA; 9 School of Medicine and Public Health, University of Wisconsin, Madison, WI 53726, USA; 10 Department of Psychiatry, Washington University School of Medicine, St Louis, MO 63110, USA; 11 Department of Medical Statistics and Bioinformatics, Leiden University Medical Center, Leiden 2300RC, The Netherlands; 12 Institut für Medizinische Biometrie und Statistik, Universität zu Lübeck, Universitätsklinikum Schleswig-Holstein, Campus Lübeck, Lübeck, Germany; 13 Department of Genetics, Texas Biomedical Research Institute, San Antonio, TX 78245, USA; 14 Department of Biostatistics, Harvard School of Public Health, Boston, MA 02115, USA; 15 Department of Laboratory Medicine and Pathology, University of Minnesota, Minneapolis, MN 55104, USA; 16 Division of Biostatistics, University of Toronto, Toronto, ON M5S 3G4, Canada; 17 Center for Inherited Disease Research, Johns Hopkins University, Baltimore, MD 21202, USA; 18 Department of Epidemiology, Emory University, Atlanta, GA 30322, USA; 19 Department of Epidemiology, University of Utah, Salt Lake City, UT 84108, USA; 20 Department of Math and Computer Science, Dordt College, Sioux Center, IA 51250, USA; 21 Department of Epidemiology and Biostatistics, Case Western Reserve University School of Medicine, Cleveland, OH 44106, USA

## Abstract

Genetic Analysis Workshop 17 (GAW17) provided a platform for evaluating existing statistical genetic methods and for developing novel methods to analyze rare variants that modulate complex traits. In this article, we present an overview of the 1000 Genomes Project exome data and simulated phenotype data that were distributed to GAW17 participants for analyses, the different issues addressed by the participants, and the process of preparation of manuscripts resulting from the discussions during the workshop.

## Introduction

This supplement of *BMC Proceedings* contains the proceedings of Genetic Analysis Workshop 17 (GAW17), which was held October 13–16, 2010, in Boston, Massachusetts, USA. The Genetic Analysis Workshops began in 1982 and are now held in even-numbered years. They provide a forum for investigators interested in identifying genetic effects on complex diseases to evaluate and compare novel and existing statistical methods. The purpose of these workshops is to allow the comparison of statistical methods for genetic epidemiology using common, well-described data sets. Before each workshop, topics are chosen, one or more existing data sets are selected, and a set of simulated data is created that permits investigation of current questions of broad interest in statistical genetics. These data are made available to any scientists who request them, and their analyses of these data are presented at the workshop. Participation in the workshop is open to anyone who submits an analysis of one of these data sets, provides data, or participates in workshop organization. More information about the Genetic Analysis Workshops, including details on upcoming meetings, can be found at http://www.gaworkshop.org.

## Genetic Analysis Workshop 17

The backdrop of GAW17 was the failure of genome-wide association studies (GWAS) to identify a set of single-nucleotide polymorphisms (SNPs) that could jointly explain a substantial proportion of the heritability in the trait for many common diseases. There is an increasing belief that the common variant/common disorder paradigm, which forms the basis for GWAS, may not be the appropriate model for describing complex disorders. An alternative paradigm is that the “missing heritability” can be explained by rare variants that cannot be identified using GWAS.

The major focus of GAW17 was the statistical challenges that arise in association analyses of exome scan data composed of real sequence information on a large number of genes from the 1000 Genomes Project and simulated phenotypes. The primary objective was to evaluate existing methods and develop novel methods to identify rare variants that modulate the phenotypes. There were two data sets: one on 697 unrelated individuals and the other on the same number of individuals distributed in 8 extended families. In the family data 202 founders were chosen at random from the set of 697 unrelated individuals. All the individuals were modeled on subjects from the 1000 Genomes Project; their genotypes were obtained from the sequence data available in that database, and their phenotypes were simulated to produce a disease trait and related quantitative risk factors influenced by multiple genes.

SNP genotypes were obtained from the sequence alignment files provided by the 1000 Genomes Project for their pilot3 study (http://www.1000genomes.org). The UnifiedGenotyper method from the Genome Analysis Toolkit (GATK) package (http://www.broadinstitute.org/gsa/wiki/index.php/The_Genome_Analysis_Toolkit) was used for the detection of SNPs and for the calling of SNP genotypes. Because the 1000 Genomes Project genotypes were not phased and because some genotypes were missing as a result of incomplete sequence coverage in some individuals, the program fastPHASE (http://depts.washington.edu/uwc4c/express-licenses/assets/fastphase/) was used to infer missing genotypes and haplotypic phase. In the family data set, the program CHRSIM [1] was used to drop the phased founder genotypes throughout the rest of the pedigree. For each of the 24,487 autosomal SNPs identified in 3,205 genes, the information provided included the chromosome and base-pair location, the name of the gene in which it was located, whether the SNP was synonymous or nonsynonymous, and the minor allele frequency. For the family data set, fully informative markers were generated at each gene and were used to compute identity-by-descent scores at each gene location.

Two hundred simulation replicates were carried out in both data sets. The genotypes were held fixed for all the replicates. Data on three quantitative phenotypes and a binary affection status phenotype were generated. Simulated data were also available on three covariates: Age, Sex, and Smoking status. A more complete description of the GAW17 data is provided by Almasy et al. [2].

The availability of the GAW17 data was announced by e-mail in the summer of 2010 to the more than 2,600 individuals on the Genetic Analysis Workshop mailing list. Two hundred four groups requested GAW17 data. One hundred sixty-six contributed papers were received that described analyses of the data sets. The GAW17 participants included 274 individuals from 19 countries: Australia, Austria, Belgium, Canada, China, Costa Rica, France, Germany, Hong Kong, India, the Netherlands, Singapore, South Korea, Spain, Switzerland, Taiwan, United Kingdom, United States, and US Virgin Islands. The 166 submitted contributions were organized into 15 presentation groups based on common methodological themes. The themes of the different presentation groups were genes with multiple rare variants (Group 1), identification of rare functional variants (Group 2), use of predicted function of gene or SNP (Group 3), identification or incorporation of gene-environment interactions (Group 4), comparison of unrelated and family data (Group 5), conditioning on known genes or variants (Group 6), scoring routines or aggregate effects (Group 7), multiple testing (Group 8), impact of linkage disequilibrium (Group 9), joint analyses of disease and risk factors (Group 10), incorporation of linkage information (Group 11), tagging of rare variants with common variants (Group 12), haplotype-based analyses (Group 13), regression and data mining methods for multiple rare variants (Group 14), and collapsing methods for rare variants (Group 15). Each presentation group was led by a person with previous Genetic Analysis Workshop experience. This person facilitated group discussions, organized the group’s oral presentation to the general meeting, and took the lead in writing the group summary paper (published in *Genetic Epidemiology*).

Members of most presentation groups began interacting before GAW17 through e-mail and a discussion forum set up on the Genetic Analysis Workshop website, comparing and contrasting their approaches and results. Each presentation group also met at least once during the workshop, where they continued their discussions and finalized a group presentation that was delivered to the full GAW17 audience during the general sessions. The group meetings were attended mostly by group participants but were open to all GAW17 attendees. During poster sessions, 87 individual contributions were presented. The 119 GAW17 contributions included in this issue of *BMC Proceedings* are a subset of the 166 contributions presented at GAW17. All these papers have been peer-reviewed and were selected on the basis of scientific merit.

The first paper in this proceedings describes the data set provided to the participants of GAW17. This is followed by the 119 individual contributions organized by presentation group and alphabetically by first author within each group. In addition, in a forthcoming supplement to the journal *Genetic Epidemiology*, a paper by each presentation group summarizes the contributions to that group and a concluding paper on the lessons learned compares and contrasts the contributions and describes their main themes and results. Overall, GAW17 generated many interesting discussions and some conclusions concerning appropriate approaches for analyzing sequence data and identifying rare causal variants. These discussions also highlighted areas in which further methodological development is needed. A general summary of these overall GAW17 conclusions is provided by Wilson and Ziegler [3].

## Competing interests

The authors declare that there is no conflict of interests.
